# A Case of Paralysis of Mortor Portion of 7th Cranial Nerve

**Published:** 1872-01

**Authors:** R. J. Preston

**Affiliations:** Abingdon, Va.


### A CASE OF PARALYSIS OF MORTOR PORTION OF 7TH CRANIAL NERVE.

BY R. J. PRESTON, OF ABINGDON, VA.

The following case was one of great interest, from the fact that the father of the child (a distinguished ex-Congressman) had died of paralysis (hemiplegia,) resulting from embolism, he having suffered from heart-disease, and nearly every member of his family, even to this little girl, being similarly affected.

S. S., a delicate little girl, aged 8 years, was seen October 30th, 1870,—being called in consultation by Dr. A. She has been suffering from acute bronchitis for some days, symptoms of which are much ameliorated and no rales scarcely to be heard over the chest—has pretty loud aortic direct murmur. Has been affected since early morning with facial paralysis—right side—involving all the mucles supplied by "Portia Dura" of 7th nerve, with all the characteristic disfigurement of the face, and some conjunctivitis of right eye and epiphora from inability to wink or close the lids. The mother of the child was much alarmed, as also was Dr. A., from the above mentioned facts taken in connection with the case. From the attendants we learned that the child had been exposed to a cold wind for a considerable part of the day before, and swelling of the face began in the night with but little pain or other disturbance. We also elicited an imperfect history of a fall, a day or two previous, and a slight bruise was still perceptible in front of right ear, below zygoma, but was not deemed important. The 3d and 6th nerves were diagnosed in tact from the absence of ptosis, strabismus, etc., and central lesion of the brain excluded from diagnosis—no previous brain symptoms having been noticed. No impairment of taste could be detected upon right side of tongue, (and the child responded intelligently and quickly to all questions,) and hence the lesion was decided to be in front of the giving off of the "Corda Tympani." Believing the paralysis entirely due to the effect of cold upon the tissues causing swelling of the same and implicating the sheath of the "Portia Dura" of 7th nerve along its extension into the long canal of petrous portion of temporal bone, thereby causing pressure upon said nerve and obliterating its functions, we gave a favorable prognosis, quieting the fears of the mother as much as possible, and ordered the following treatment: The child's bowels to be kept open by the use of gentle laxatives—blisters to be applied behind right ear as often as needed, to keep up counter-irritation, and affected muscles to be rubbed with lint. ammonia frequently. Hot pediluvia to be given every night, the child to be kept within doors and right eye covered to protect it from the light.

The results of this treatment confirmed the diagnosis, as marked improvement followed it, the child being perfectly recovered in ten days, and all the serious apprehensions of the mother and her physician entirely dissipated.

